# White matter microstructure alterations in primary dysmenorrhea assessed by diffusion tensor imaging

**DOI:** 10.1038/srep25836

**Published:** 2016-05-10

**Authors:** Peng Liu, Geliang Wang, Yanfei Liu, Qingbao Yu, Fan Yang, Lingmin Jin, Jinbo Sun, Xuejuan Yang, Wei Qin, Vince D. Calhoun

**Affiliations:** 1 Life Science Research Center, School of Life Science and Technology, Xidian University, Xi’an 710071, China; 2 The Mind Research Network, Albuquerque, New Mexico 87106, USA; 3 Department of Electrical and Computer Engineering, University of New Mexico, Albuquerque, New Mexico 87131, USA

## Abstract

Primary dysmenorrhea (PDM), a significant public health problem for adolescents and young women, is characterized by painful menstrual cramps. Recent neuroimaging studies have revealed that brain functional and structural abnormalities are related to the pathomechanism of PDM. However, it is not clear whether there are white matter (WM) alterations in PDM. We analyzed diffusion tensor imaging data from 35 patients and 35 healthy controls (HCs) matched for age and handedness. Tract-based spatial statistics and probabilistic tractography were used to measure integrity of WM microstructure. Compared to HCs, patients had increased fractional anisotropy (FA) along with decreased mean diffusivity (MD) and radial diffusivity (RD) in the corpus callosum (CC), superior longitudinal fasciculus (LF), corona radiata (CR), internal capsule (IC) and external capsule (EC). The FA of the splenium CC and right IC positively correlated with PDM duration while FA of the right anterior CR positively correlated with PDM severity in patient group. These WM tracts were found to show connections to other brain regions implicated in sensoimotor, affective, cognitive and pain processing functions through tractography. These findings provide preliminary evidence for WM microstructure alterations in PDM, which is potentially valuable for understanding pathomechanism of PDM.

As a significant public health problem for adolescents and young women, primary dysmenorrhea (PDM) is characterized by painful menstrual cramps of uterine origin in the absence of pelvic pathology and enhanced pain sensitivity^1^,^2^,^3^. PDM is prevalent in 20% to 90% of female adolescents^4^, has a negative effect on quality of life for women, and results in tremendous health care costs^5^,^6^. Previous studies have proposed that PDM is associated with prostaglandins and painleukotrienes, which may play a role in modulating hyperalgesia and inflammatory pain and causing uterine contractions^4^,^6^. However, the clear mechanism underlying PDM remains to be determined.

Recent functional brain imaging studies have provided evidence to support the idea that abnormal brain functions may contribute to developing or maintaining PDM^7^,^8^. For example, PDM patients had abnormalities of cerebral metabolism in the nociceptive and pain modulatory pathways, including increased metabolism in the prefrontal cortex (PFC), orbitofrontal cortex (OFC), thalamus and decreased metabolism in the sensorimotor cortex^7^. Given thermal noxious stimulation, PDM patients exhibited altered regional activations linked to the hypothalamic–pituitary–adrenal (HPA) axis and central sensitization^8^. Meanwhile, structural brain imaging studies have enabled us to characterize alterations of brain anatomical substrates in PDM^9^,^10^,^11^. Tu and colleagues found that PDM patients had increased volumes of gray matter (GM) were mainly located in the anterior/posterior cingulate cortex (ACC/PCC), hippocampus (HIPP), hypothalamus and midbrain, As well as decreased ones were located in the PFC, OFC, insula and somatosensory cortex^10^. In a previous study, we reported increased cortical thickness in the OFC, insula, primary/secondary sensory area (SI/SII), superior temporal cortex, precuneus and PCC, as well as decreased subcortical volumes in the caudate, thalamus and amygdala in PDM patients^9^. Moreover, changes of GM were reported to be associated with the states of dysmenorrhea (the dysmenorrhea pain and pain-free states)^11^. These aforementioned studies suggest that PDM is accompanied by central nervous system (CNS) abnormalities which may predispose to chronic pain.

To better understand structural and/or functional characteristics, the microstructural properties of white matter (WM) should be investigated. Investigating relationships between certain disorder and changes of WM microstructure could further provide potential value for understanding the pathomechanism of the disorder. Diffusion tensor imaging (DTI) is a novel technique to assess WM microstructure and/or connectivity *in vivo*^12^,^13^. DTI metrics include fractional anisotropy (FA), mean diffusivity (MD), radial diffusivity (RD) and axial diffusivity (AD). FA reflects WM integrity or directionality of the molecular motion of water^14^,^15^. MD is associated with edema and inflammation^16^. RD possibly reflects changes in membrane permeability and myelination^17^. AD is related to diffusion along tracts. FA and other diffusivity measures have been used to examine changes in WM microstructural pathways involved in pain processing^18^. However, little is known regarding whether there are altered WM microstructures in PDM patients.

In this study, we tried to evaluate changes of WM and reorganization of the WM connectivity in PDM patients using tract-based spatial statistics (TBSS)^19^ and probabilistic tractography^20^, and to determine whether parameters of the abnormalities in the WM tracts correlated with clinical characteristics of PDM. Based on previous studies, we hypothesized that alterations of WM would be found in the tracts associated with pain processing, sensory, motor, affection and cognition. We also expected that clinical characteristics would correlate with parameters of tracts that are related to interoception and affective processing of pain. In addition, psychosocial factors, such as depression and anxiety, are known to be associated with menstrual pain^21^,^22^ and exert their role in pain processing in other pain disorders^23^,^24^,^25^,^26^. Thereby, we further hypothesized that psychosocial factors might influence on alterations of WM in PDM.

## Results

### Demographic and clinical characteristics

The demographic and clinical characteristics of all subjects are shown in Table 1. Demographic characteristics between 35 PDM patients and 35 healthy controls (HCs) were not significantly different, including age, body mass index (BMI), onset of menses and length of menstrual phase (*P* > 0.05). However, there were significantly higher McGill pain questionnaire (MPQ)^27^,^28^, Cox retrospective symptom scale (RSS)^29^, self-rating anxiety scale (SAS)^30^ and self-rating depression scale (SDS)^31^ in PDM patients compared with HCs (*P* < 0.005).

### White matter microstructure alterations

**Figure 1:**
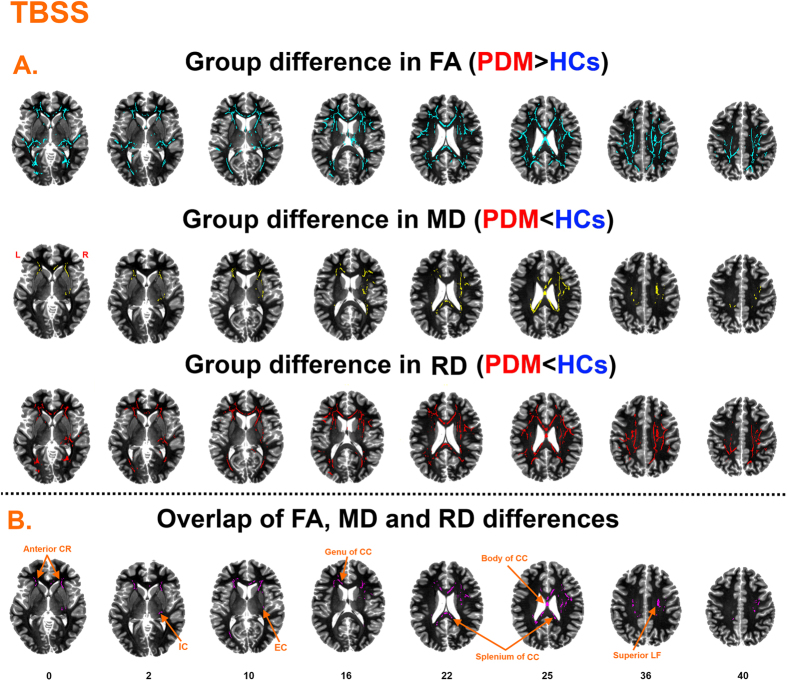
TBSS findings. (**A**) Regions showing increased FA (Fig. 2A first row), decrease MD (Fig. 2A second row) and decreased RD (Fig. 2A third row) in PDM patients. (**B**) Overlapping regions including the anterior corona radiata (anterior CR), internal capsule (lC), corpus callosum (genu, body and splenium of CC), external capsule (EC), superior longitudinal fasciculus (superior LF).

**Figure 2:**
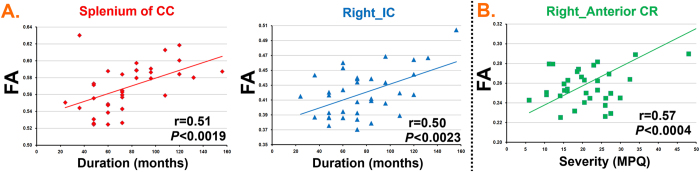
Overlapping regions correlated with clinical characteristics of primary dysmenorrhea (PDM). (**A**) The FA of splenium of corpus callosum (CC) and right internal capsule (lC) positively correlated with PDM duration. (**B**) The FA of right anterior corona radiata (CR) positively correlated with PDM severity.

Compared with HCs, PDM patients had significantly increased FA, decreased MD and RD in many regions shown in Fig. 1A. Specifically, PDM patients had increased FA mainly in tracts of the corpus callosum (CC) (genu, body and splenium), fornix, bilateral internal capsule (IC), bilateral external capsule (EC), corona radiata (CR), bilateral posterior thalamic radiation, bilateral sagittal stratum, right cingulum and bilateral superior longitudinal fasciculus (LF). Decreased MD and RD in PDM patients was mainly obsered in the bilateral CC, right IC, bilateral CR, right EC and right superior LF. The overlapping regions exhibiting increased FA and decreased MD and RD included the CC (genu, body and splenium), right superior LF, bilateral anterior CR, right IC and right EC (Fig. 1B and Table 2). In these regions, FA of overlapping regions correlated with PDM clinical characteristics including duration and severity (MPQ). In detail, the FA of splenium CC and right IC positively correlated with PDM duration (Fig. 2A). The FA of the right anterior CR positively correlated with PDM severity (Fig. 2B). Results were considered significant when passing *P* < 0.05/8 = 0.00625.

**Figure 3:**
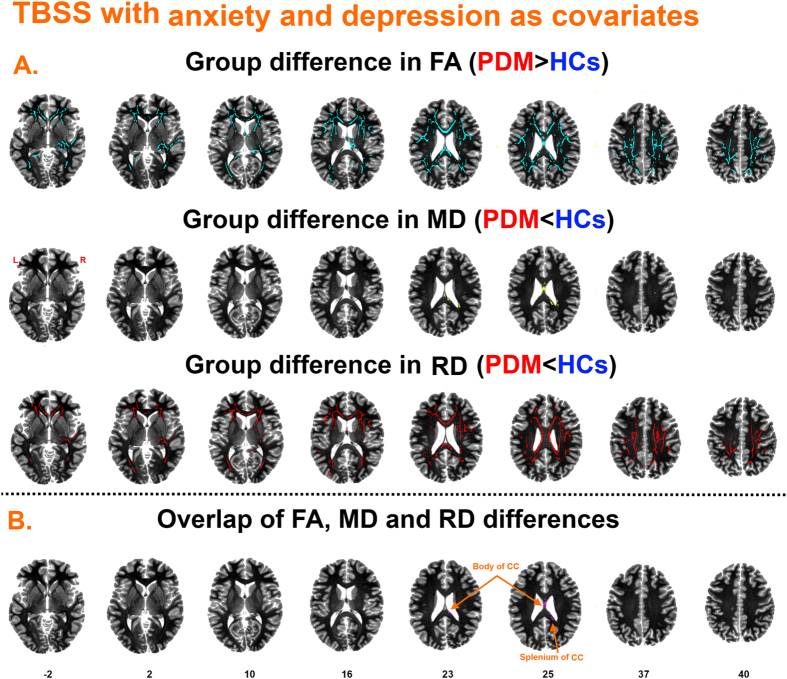
TBSS findings with self-rating anxiety scale (SAS) and self-rating depression scale (SDS) as covariates. (**A**) Regions showing increased FA (Fig. 3A first row), decreased MD (Fig. 3A second row) and decreased RD (Fig. 3A third row) in PDM patients compared to healthy controls. (**B**) Overlapping regions including the body and splenium of corpus callosum (CC).

TBSS results with anxiety and depression as covariates of no interest are shown in Fig. 3 and Table 2. The FA and RD maps showed moderate changes, while the MD maps showed almost no changes. Overlapping significant regions for FA, MD and RD were found in the CC (body and splenium).

### Probabilistic tractography

**Figure 4:**
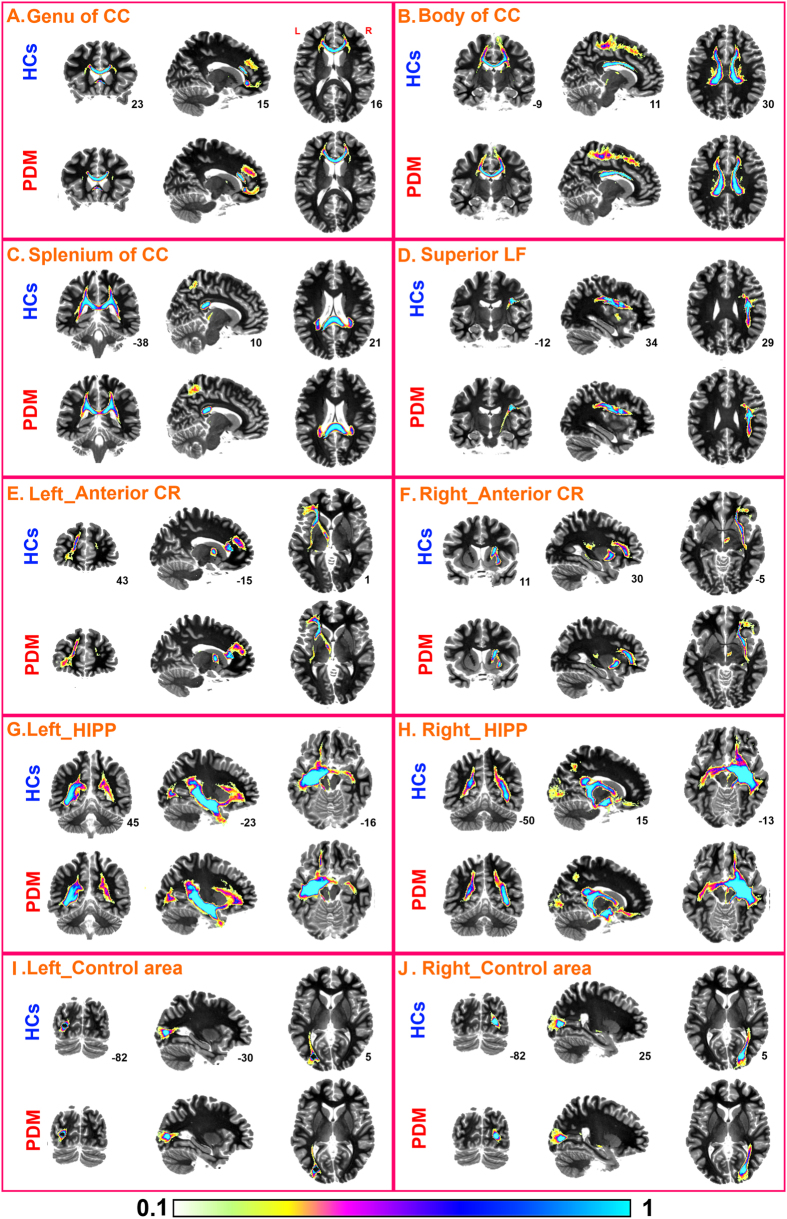
Probabilistic tractography of seed regions in both PDM patients and healthy controls, including the genu, body, splenium of corpus callosum (CC), the superior longitudinal fasciculus (superior LF), anterior corona radiata (CR), hippocampus (HIPP) and two control areas in visual cortex.

Tractography analysis was performed in the patient and control groups respectively, using the identified group-differentiating regions (genu, body, splenium of CC, right superior LF and anterior CR) as seed regions (Fig. 4). Through visual inspection, PDM patients appeared to have denser connections between the genu of CC and medial prefrontal cortex (MPFC) (Fig. 4A); between the body of CC and supplementary motor cortex, somatosensory cortex, middle cingulate cortex (MCC) and thalamus (Fig. 4B); and between the splenium of CC and precuneus (Fig. 4C) compared to HCs. And denser connections in PDM patients were also noted between the superior LF and putamen, insula, hippocampus and amygdala (Fig. 4D); and between anterior CR and MPFC, lateral prefrontal cortex and ACC (Fig. 4E,F).

Furthermore, we also investigated the WM tracts through HIPP, as it is well documented that HIPP is involved in PDM^10^. Compared to HCs, PDM patients had denser connections between the HIPP and MPFC, amygdala and OFC (Fig. 4E,F).

Finally, we selected two control regions in the visual cortex that is likely not related to PDM. We used these two regions as seed regions and performed the tractography analysis. Overall the connectivity patterns of these tracts were largely comparable between the two groups (Fig. 4I,J).

## Discussion

To the best of our knowledge, this was the first study to investigate WM microstructure alterations in PDM patients by diffusion-weighted MRI. We found that a) PDM patients had increased FA, decreased MD and RD in various WM tracts compared to HCs, mainly including the CC, superior LF, anterior CR, IC and EC; b) the FA of the splenium CC and right IC positively correlated with PDM duration, and the FA of the right anterior CR positively correlated with PDM severity; c) there were abnormal connectivity patterns of the genu, body and splenium of CC, superior LF, anterior CR and HIPP with other brain regions implicated in sensory, motor, cognitive, affective and pain processing; c) the WM microstructure alterations might be partly related to psychosocial factors in PDM.

The first finding in the current study was that PDM patients had increased FA in the main tracts including the fornix, IC, EC, CR, CC, superior LF and thalamic radiation and the tracts extended to the OFC, PFC, insula, parietal cortex, cingulate cortex and basal ganglia. In most of the previous pain studies, decreased FA was reported to be associated with chronic pain, such as temporomandibular disorder^32^, fibromyalgia^33^, back pain^34^ and complex regional pain syndrome^35^. However, increased FA-related findings have been observed in other pain conditions^36^,^37^. For example, Chen *et al.* found that patients with irritable bowel syndrome (IBS) had increased FA in the fornix, EC, IC, insula, thalamus, cingulate cortex and SI^36^. Recently, we found increased FA in the EC, IC, CC, LF and CR^37^ in patients with postprandial distress syndrome. Moreover, it was reported that repeated juggling induced used-dependent plastic changes in the WM with increased FA^38^. Taken together, our findings could indicate that via plastically learning process from nociceptive input with menstruation, PDM patients might have increased myelination, increased number of myelination fibers or increased ratio between longitudinal vs. oblique aligned myelinated fibers in WM tracts. And the altered WM microstructures could be related to specific characteristic of PDM rather than the chronic painful state, which should be further evaluated in future studies.

In this study, PDM patients were found to have increased FA in the genu, body and slpenium of CC along with lower MD and RD, and had altered connectivity of the subregions of CC to the MPFC, supplementary motor cortex, somatosensory cortex, middle anterior/middle cingulate cortex, thalamus and precuneus in this study. CC, the largest commissural white matter bundle in brain, is topographically organized with its genu connecting orbitofrontal and frontal cortices, while its body and splenium connecting temporal, parietal and occipital regions^39^,^40^. Playing an important role in interhemispheric communication, CC is also critical to the evolution of structures and functions of cerebral cortex^41^. Neuroimaging studies have identified that PDM patients have dysfunctional nociceptive pathways and pain modulatory pathway and sensorimotor regions including the PFC, OFC, thalamus, insula and sensorimotor cortex^7^,^10^. Our findings might suggest that PDM patients have abnormalities in integrating pain modulation and sensory information which could relate to the disrupted structural connectivity arising from white matter microstructural anomalies in the CC. Furthermore, it has been revealed that abnormal interhemispheric transfer in the CC contributes to heightening pain perception and strengthening augmented pain processing^42^, which could be an interpretation of enhanced responses to noxious stimuli in PDM condition. On the other hand, fibers in splenium of CC connect regions of the parietal cortex involved in somatosensory information processing^43^. The positive correlation between FA of the splenium of CC and PDM duration in our study could reflect abnormal processing of somatosensory and pain as a consequence of extended periods of PDM.

Another finding of this study is that PDM patients showed altered WM microstructure in the superior LF. Superior LF is a complex brain association fiber system connecting the front and the back of the cerebrum including frontal, parietal and occipital cortex. It originates from the frontal cortex, passes through the operculum and ends in the posterior area of the lateral sulcus where numerous neurons radiate into the occipital cortex while others turn downward and forward around the putamen and radiate to anterior temporal cortex^44^. WM changes in the superior LF have been reported in pain-related disorders, implicated in the cognitive-affective dimension of pain, attention and somatosensory input^37^,^45^,^46^. In our study, PDM patients had denser connections between the superior LF and brain regions including hippocampus, amygdala, putamen and insula. Our findings may suggest that altered WM microstructures in the superior LF are likely involved in abnormalities of cognitive and emotional processing in PDM patients.

PDM patients had WM microstructure alterations in the IC. IC conveys information from primary and supplementary motor areas, frontopontine and thalamic peduncles to brain stem and cerebellar regions, and from thalamus to prefrontal cortex^47^. The damage in the posterior limb of IC, adjacent to the sensory thalamus, can produce ataxia^48^. Stimulation of the posterior limb of IC also may evoke paresthesias^49^. In addition, PDM patients had WM microstructure alterations in the EC adjacent to the posterior insula in this study. Craig provides a model of interoceptive input such that interoceptive information regarding the physiological condition of the body is projected to the anterior insula via the posterior insula^50^. The involvement of the posterior insula in visceral regulation and in homeostatic regulation is well elaborated^51^. EC also conveys information pertaining to the emotional component of pain perception^46^. Thereby, we speculate that WM microstructure alterations in the IC and EC might be implicated in pain and abnormal sensation arising from uterine, which could yield abnormal hypometabolisms in the sensorimotor and/or emotional regions^7^. Considering the positive correlation between the IC and PDM duration, we further speculate that WM microstructure alterations in the IC might be driven by extended periods of PDM.

We found WM microstructure alterations in the anterior CR in PDM patients. Anterior CR includes thalamic projections from the internal capsule to the cortex, and is a part of limbic-thalamo-cortical circuitry which is implicated in emotional regulation (Drevets *et al.*, 2008). It has been reported that during menstruation women with PDM are more agitated and have poorer mood states, which are strongly associated with PDM^52^,^53^. Tu *et al* suggested that disinhibition of thalamo-orbitofrontal-prefrontal networks could contribute to the generation of pain and increased pain sensitivity in PDM patients, possibly by increasing negative emotion^7^. Additionally, anterior CR connects the ACC and striatum^54^. Altered gray matters in the ACC and striatum have been associated with PDM^9^,^10^. Abnormal activation or structure in the ACC is often found in experimental pain and chronic pain, and is related to affective aspects of pain processing^55^. Striatum is also enrolled in motor and emotional processing^56^,^57^,^58^. Thereby, our findings might indicate that PDM patients have abnormal processing of emotion which could associate with WM changes in the anterior CR.

PDM patients had abnormal connectivity between the HIPP and brain regions including the MPFC, amygdala and OFC in this study. HIPP is known to be a prime target for memory and learned behavior. However, it is also involved in inhibitory feedback to the HPA axis by stress^59^. Amygdala has an important direct influence on the emotional dimension of pain as well as fear, anxiety, and depression. There is a classic fear learning circuit related to amygdala and hippocampus^60^. HIPP and MPFC cooperate during anxiety processing as well^61^. Thereby, our finding might suggest that PDM may be sensitive to stress or negative emotion. Abnormal hippocampal structural connectivity might be hypothesized as a generating mechanism in PDM.

Increasingly, psychosocial factors such as anxiety, depression and catastrophizing have proven to be important contributors to patients with pain disorders^23^,^24^,^25^,^26^. As early as the late 1970s, the relationship between psychological factors and severity of dysmenorrhea was reported^62^. Our results with anxiety and depression as covariates showed that the tracts showing WM microstructural alterations only included the body and splenium of CC (Fig. 3). Our findings support that psychosocial factors partially influence on WM microstructure in PDM.

The present study presented some limitations which should be worth mentioning: a) WM changes near a structure are not necessarily attributable to axonal changes. These are huge axonal bundles that cross large stretches of brain, and abnormal axonal segment may just be passing through the neighborhood. Without T1 results to correlate volume/density with local white matter changes, we really cannot verify whether these FA changes are relevant to the described brain regions. Multimodal fusion of brain imaging data should be used in the future to further assess neural mechanisms of PDM. b) The number of collected diffusion directions was 30 in this study. Neuroimaging data with more number of diffusion directions should be collected to retest the current results. c) The DTI data were collected from PDM patients, who were not in the menstrual cycle. It has been found that PDM-related changes of GM structure were related with the dysmenorrhea pain or pain-free states^11^. So, our future study can be focused more on the question: whether there are differences of WM between the different sates of the PDM patients.

## Conclusion

In summary, to the best of our knowledge, we provide the first evidence that PDM patients have white matter microstructure alterations, abnormal structure connectivity and that the PDM clinical characteristics were related to altered white matter tracts. To a certain extent, the psychology factors also disrupted pattern of white matter microstructure in PDM patients. These findings might contribute to our understanding of the neural mechanism of PDM. However, the interaction between the altered white matter microstructures and PDM should be more comprehensively investigated in future longitudinal studies and in a larger population of PDM patients.

## Methods

All research procedures of the present study were conducted in accordance with the Declaration of Helsinki and were approved by the West China Hospital Subcommittee on Human Studies. Informed consent was obtained from all subjects before participation. And all methods were carried out in accordance with the approved guidelines.

### Subjects

35 right-handed PDM patients and 35 age-matched right-handed healthy females were recruited in this study. All the subjects were university students from Chengdu University of Traditional Chinese Medicine or West China School of Medicine of Sichuan University. Patients were included if they met all of the following inclusion criteria: a) regular menstrual cycle (28 ± 3days) and menstruation lasting 3 to 7 days; b) moderate or severe dysmenorrhea (based on numeric rating scales (NRSs), greater than 4 points^7^ on a visual analogue scale in which 0 means no pain and 10 means worst pain imaginable); c) attacking at least 4 months in the past 6 months and being nulliparous. Exclusion criteria for all the subjects included: a) secondary dysmenorrhea induced by endometriosis or pelvic inflammatory disease; b) recurrent pelvic or lower abdominal pain; c) gastrointestinal diseases including recurrent gastritis, gastric or duodenal ulcer; d) chronic pain; e) being pregnant or intending to become pregnant during the course of the trial; f) having history or evidence of serious diseases, neurological or psychiatric diseases; g) any contraindication of MRI scanning; h) receiving oral contraceptives within 6 months prior to this study; i) smokers; j) heavy drinker or alcohol/drug addicts; k) receiving NSAIDs (including aspirin) or analgesics within 24 h before MRI scanning. In addition, each patient underwent a basic evaluation with pelvic ultrasound to further ensure to exclude PDM patients caused by endometriosis, uterine myomas, endometrial polyps, pelvic inflammatory disease, and other gynecological problems. Healthy female controls also underwent the same basic evaluation mentioned above.

### Clinical assessment

McGill pain questionnaire^27^,^28^ and Cox retrospective symptom scale^29^ were applied to assess the lower abdominal menstrual pain and associated symptoms of each PDM patient in past three months. self-rating anxiety^30^ and self-rating depression^31^ scales were then adopted to evaluate subject’s anxiety and depression status. Other baseline data including age, body mass, height, length of menstrual cycle, length of menstrual phase and duration of dysmenorrhea were recorded as well. All the related assessments were completed before scanning, similar to the previous study from Tu *et al.*^7^,^10^.

### Scanning protocol

All the subjects underwent scanning in a 3T Siemens scanner (Allegra, Siemens Medical System, Erlangen, Germany) at the Huaxi MR Research Center, West China Hospital of Sichuan University, Chengdu, China. Two Diffusion-weighted sequences with single-shot echo planar imaging in alignment with the anterior–posterior commissural plane were acquired with the following parameters: repetition time (TR) = 6800 ms, echo time (TE) = 93 ms, field of view (FOV) = 240 mm × 240 mm, matrix = 128 × 128, 1.875 mm × 1.875 mm in-plane resolution, slice in thickness = 3 mm, 45 continuous axial slices with no gap, b = 1,000 seconds/mm^2^, diffusion sensitizing gradients applied along 30 non-collinear isotropic directions, with two no diffusion weighting image (b0 = 0 seconds/mm^2^).

### Demographic and clinical characteristics analysis

Continuous variables of demographic, clinical symptom severity, and emotional measures were tested by an independent 2-sample t-test. Statistical significance was set to *P* < 0.05 (two-tailed test).

### DTI image analysis

Because DTI data may be disturbed by cardiac pulsatility^63^, signal dropout, motion artifacts and/or other artifacts, all the DTI image volumes were visually checked before preprocessing to screen noisy images that could bias the results. We also inspected data for volumes containing large signal variation or ghosting.

The diffusion image processing was performed using the Oxford Centre for Functional MRI of the Brain’s (FMRIB) Software Library (FSL version 4.1.6, http://www.fmrib.ox.ac.uk/fsl)^64^. Preprocessing included eddy current and motion artifact correction using the FSL diffusion toolbox (FDT)^65^. The mean DTI images were calculated from the two preprocessed DTI acquisitions for each subject for a greater signal-to-noise ratio. The preprocessed images were then fit with a diffusion tensor model using DTIFIT in the FMRIB diffusion toolbox after individual brain masks were created from the b0 image of each subject using the by brain extraction tool^66^. FA, MD, RD and AD maps were generated from the duffusion tensor model.

Voxelwise statistical analysis of FA data was carried out by TBSS^19^. Individual FA images underwent nonlinear registration to a standard space (a 1-mm isotropic FA image (FMRIB58_FA)) using the non-linear registration tool FNIRT. A mean FA image stemmed from all the subjects’ images were created and thinned to represent the center of major WM tracts common to all subjects, forming a mean WM skeleton with an FA threshold of >0.25. Each subject’s aligned FA map was projected onto the nearest relevant tract center of the mean FA skeleton by searching perpendicular to the local skeleton structure, which circumvents misalignment during registration. One skeleton was created for analysis of group differences that included all the subjects. Combing FA, MD, RD and AD would probably provide more information about the neural mechanism of PDM. We thereby assessed the other DTI metrics similar to the analysis steps of FA.

FSL’s permutation-based non-parametric testing (Randomise v2.1) was then adopted to compare FA between the patient and HCs with 10,000 times. Multiple comparisons across voxels were corrected using the threshold-free cluster enhancement (TFCE) method at *P* < 0.05, with a cluster size of >100 voxels^67^. Other metric images were also examined using TBSS, related to MD, RD and AD. The WM tracts with significant difference were identified by the JHU ICBM-DTI-81 white-matter label atlas^68^.

To investigate relationships between parameters of WM tracts and clinical characteristics, Pearson correlation analysis was adopted to examine relationships between PDM clinical characteristics (duration and severity) and FA of the WM tracts showing between-group differences in the overlapping map. The significance level was set at *P* < 0.05/N for multiple tests (Bonferroni-corrected, where N is the number of WM tracts tested).

We selected seed regions including the WM tracts showing between-group differences in DTI measures, HIPP and visual cortex as control areas. Bilateral HIPP were defined from the Harvard–Oxford Structural Probability Atlas distributed with the FSL neuroimaging analysis software package. Seed regions of HIPP was thresholded at 75% to yield a conservative anatomical representation. Seed regions of visual cortex were spheres centered on MNI coordinates of sphere: ±27, −80, 6 and radius = 4 mm, that were related to PDM. Probabilistic tractography was then adopted to examine connectivity of these seed regions, using FDT. By fitting a multifiber diffusion model, the probability distribution on direction of 1 or more fiber populations at each voxel was assessed for the pathways through regions of fiber crossings^69^. The connectivity distribution images, in which each voxel represented the probability of the connection to the seed voxel, were built up by producing 5,000 streamline samples from each seed voxel with parameters of curvature threshold at 0.2 and step length at 0.5 mm. Seed masks were binary significant clusters identified with TBSS analysis. The connectivity distribution of each subject was thresholded at 2,500 to remove spurious connections^37^,^70^,^71^. To visualize the group findings, each of the subject’s tracts were binarized and overlaid on a standard brain to produce a probabilistic map of the pathways for patients and HCs, respectively.

In order to investigate the effects of psychosocial factors on DTI-derived metrics, we carried out TBSS between the two groups with anxiety and depression as covariates. Multiple comparisons were also corrected using the TFCE at *P* < 0.05.

## Additional Information

**How to cite this article**: Liu, P. *et al.* White matter microstructure alterations in primary dysmenorrhea assessed by diffusion tensor imaging. *Sci. Rep.* **6**, 25836; doi: 10.1038/srep25836 (2016).

## Figures and Tables

**Table 1:** Comparison of demographics and clinical symptoms between PDM patients and healthy controls.

Items	PDM (Mean ± SD)	HCs (Mean ± SD)	*p*-value
Age(year)	22.6 ± 1.1	22.3 ± 1.8	–
Body mass index	21.8 ± 1.8	21.7 ± 1.6	–
Onset of menses (year)	12.5 ± 1.2	12.7 ± 1.3	–
Length of menstrual phase (day)	3–7	3–7	–
Duration of dysmenorrhea (year)	6.3 ± 2.5	–	–
MPQ	22.0 ± 9.6	0	<0.001
RSS	34.6 ± 16.8	5.7 ± 2.1	<0.001
SAS	30.1 ± 5.9	25.2 ± 6.6	<0.005
SDS	31.6 ± 8.5	25.4 ± 5.5	<0.001

Abbreviations: SD, standard deviation; PDM, primary dysmenorrheal; HCs, healthy controls; RSS, Cox retrospective symptom scale; MPQ, McGill pain questionnaire; SAS, self-rating anxiety scale; SDS, self-rating depression scale.

**Table 2:** Main locations of white-matter tracts showing differences of FA, MD and/or RD in PDM patients compared to healthy controls.

White-matter tracts	none			anxiety and depression		
	FA	MD	RD	FA	MD	RD
Genu of corpus callosum	1137	371	1107	1021		838
Body of corpus callosum	2852	1246	2713	2842	219	2517
Splenium of corpus callosum	605	627	646	406	163	573
Internal capsule R	791	122	570	740		167
Internal capsule L	833		191	722		342
Anterior corona radiata R	1463	542	1357	1360		1184
Anterior corona radiata L	1497	538	1398	1430		1291
Superior corona radiata R	924	717	1120	955		918
Superior corona radiata L	521	191	463	523		353
Posterior corona radiata R	459	392	581	538	217	350
Posterior corona radiata L	419	293	421	419		361
Posterior thalamic radiation R	555		345	527		
Posterior thalamic radiation L	449	113	378	413		
Sagittal stratum R	252		207	221		
Sagittal stratum L	317					
External capsule R	116	226	326			213
External capsule L	142			305		
Cingulum (cingulate gyrus) R	145			118		
Fornix R	151					
Fornix L	187					
Superior longitudinal fasciculus R	921	253	845	824		471
Superior longitudinal fasciculus L	774		595	681		339

The labels of the columns indicated the factors used as covariates.

Abbreviations: FA, fractional anisotropy; MD, mean diffusivity; RD, radial diffusivity; L, Left; R, Right.
